# Supplementary material for: The conformational wave in capsaicin activation of transient receptor potential vanilloid 1 ion channel
Source: Nat Commun. 2018 Jul 23;9:2879. doi: 10.1038/s41467-018-05339-6 (PMC6056546; doi:10.1038/s41467-018-05339-6)
Supplement: Supplementary file 1 — Supplementary Information [file 41467_2018_5339_MOESM1_ESM.pdf]

The conformational wave in capsaicin activation of Transient Receptor Potential Vanilloid 1 ion channel

Fan Yang[1,3#¶], Xian Xiao[2,3#], Bo Hyun Lee[3#§], Simon Vu[3], Wei Yang[1], Vladimir Yarov-Yarovoy[3] and Jie Zheng[3¶]

[1]Department of Biophysics and Kidney Disease Center, First Affiliated Hospital, Institute of Neuroscience, National Health Commission and Chinese Academy of Medical Sciences Key Laboratory of Medical Neurobiology, Zhejiang University School of Medicine, Hangzhou 310058, Zhejiang Province, China. [2]Institute for Basic Medical Sciences, Westlake Institute for Advanced Study, Westlake University, Shilongshan Road No. 18, Xihu District, Hangzhou 310024, Zhejiang Province, China. [3]Department of Physiology and Membrane Biology, University of California, Davis, California 95616, USA.

[#] These authors contributed equally to this work.

[§] Current address: University of Washington, Department of Physiology and Biophysics, Seattle WA 98195

[¶] Correspondence and requests for materials should be addressed to F.Y. (email: fanyanga@zju.edu.cn) or to J.Z. (email: jzheng@ucdavis.edu)

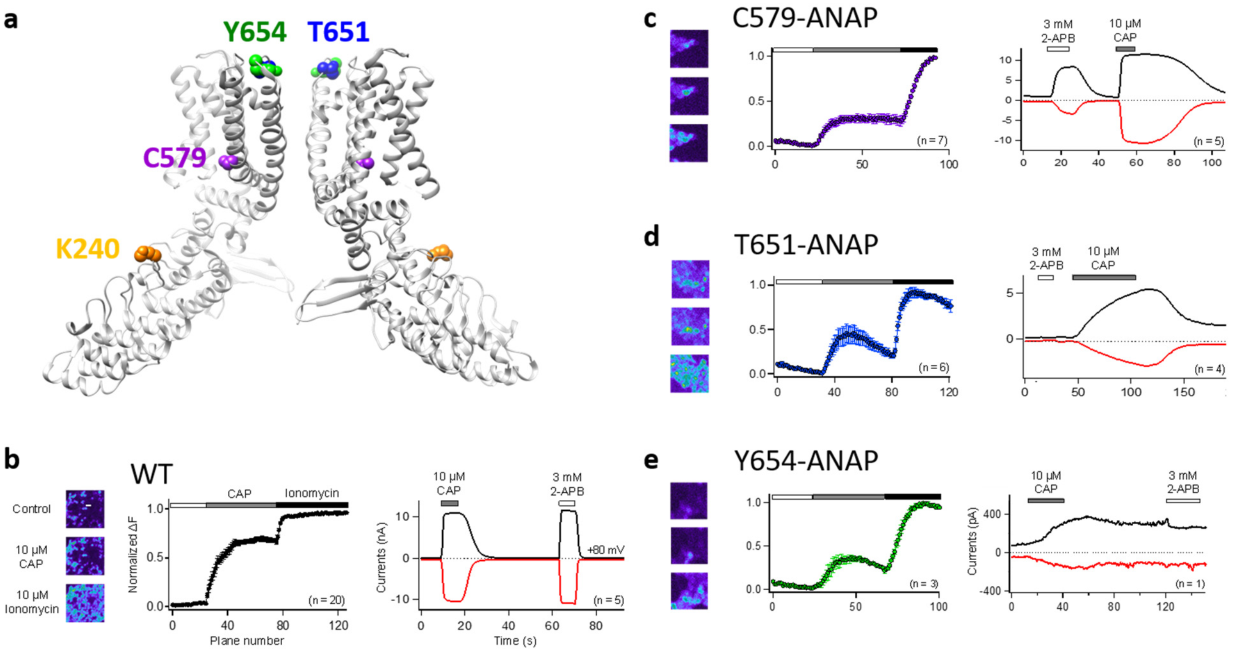

Supplementary Figure 1. Functional confirmation of ANAP-incorporated TRPV1 channels. (**a**) Location of ANAP-incorporated sites. (**b to e**) In calcium imaging, 10 µM capsaicin was used to test the function of channels expressed in HEK293T cells. 3 mM ionomycin was perfused at the end of imaging as a positive control. In whole-cell recordings, 10 µM capsaicin and 3 mM 2-APB were perfused. Channels with ANAP incorporated at C579, T651 and Y654 site were activated by capsaicin in both calcium imaging and whole-cell recording. The scale bar: 100 µm.

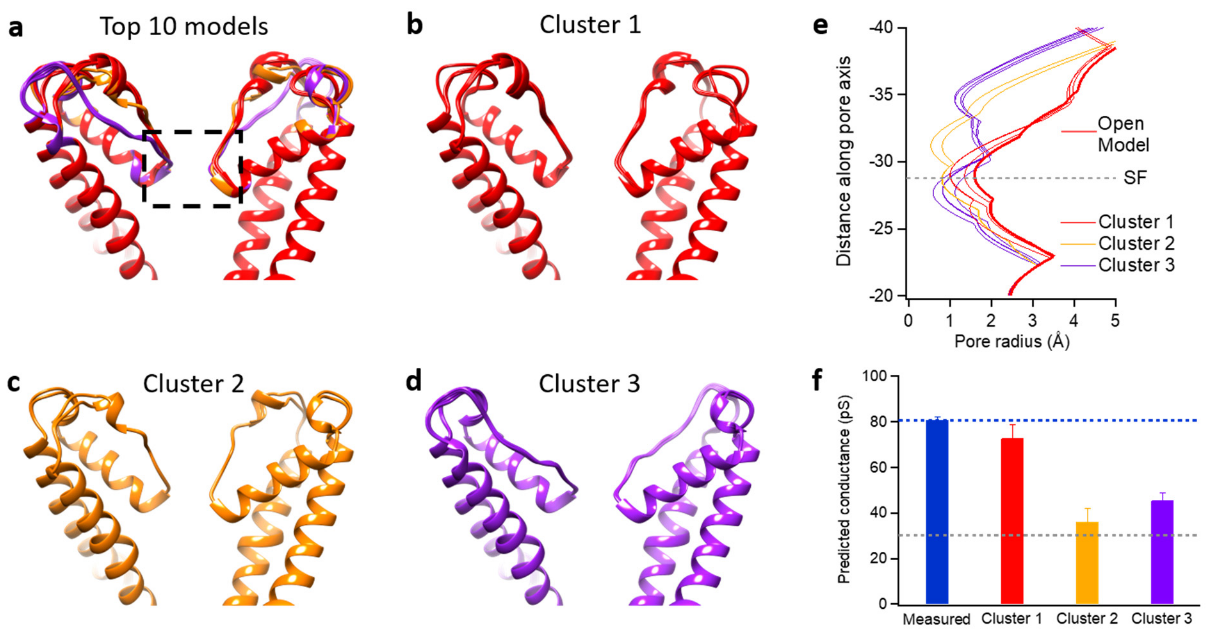

Supplementary Figure 2. Clusters of the top 10 open state models. (**a**) Top 10 models of capsaicin-induced open state channel. The selectivity filter was boxed in dashed line. (**b** to **d**) Cluster 1 to 3 were shown, respectively. (**e**) Distribution of pore radii in Cluster 1 to 3. (**f**) Predicted conductance of the models in different Clusters.

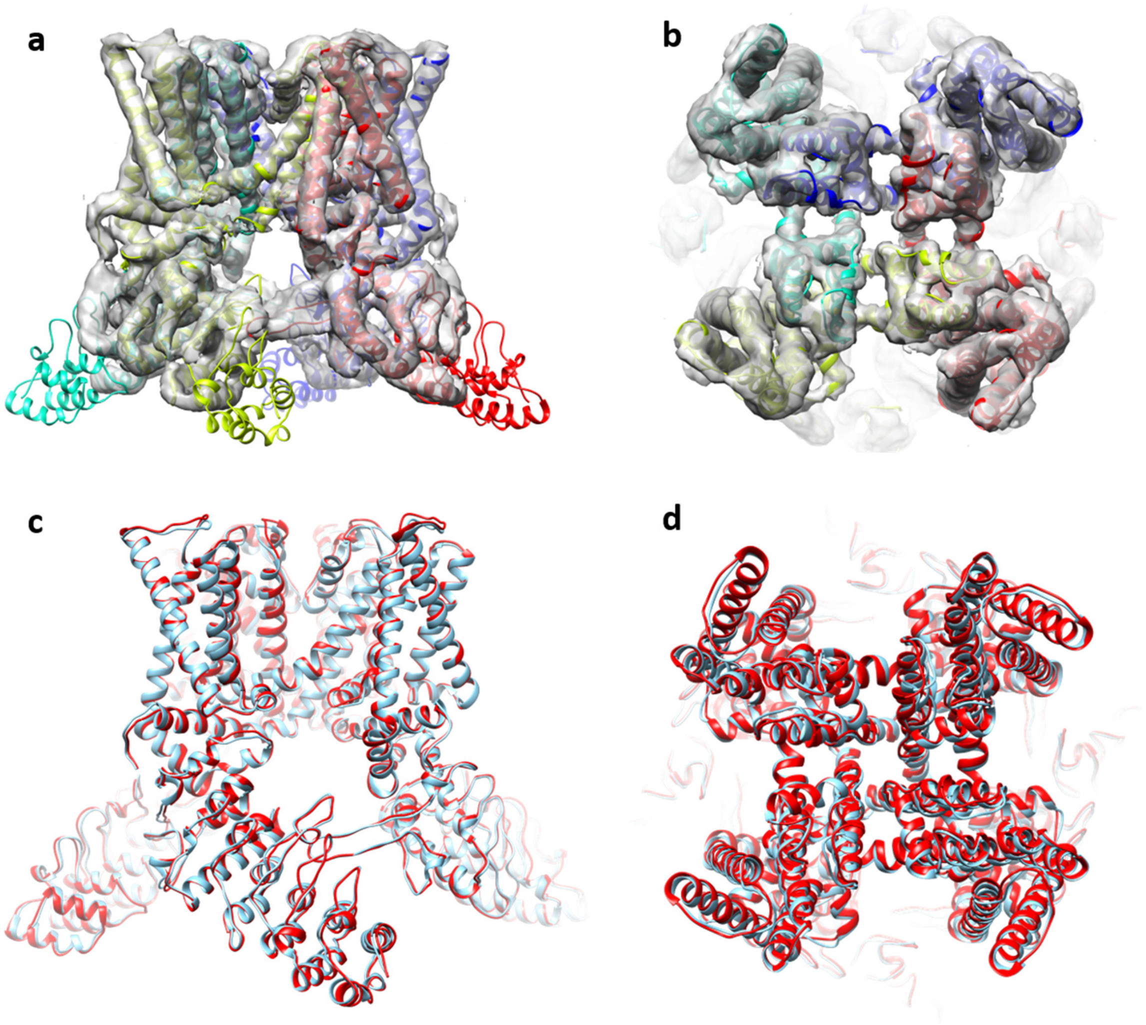

Supplementary Figure 3. Comparison between our refined open state model and cryo-EM structure models. (**a** and **b**) Side and top view of our refined open state model fitted into the cryo-EM density map (capsaicin-bound state, EMD ID: 5777). (**c** and **d**) Side of top view of our refined open state model (blue) aligned with the open state model before refinement (red).

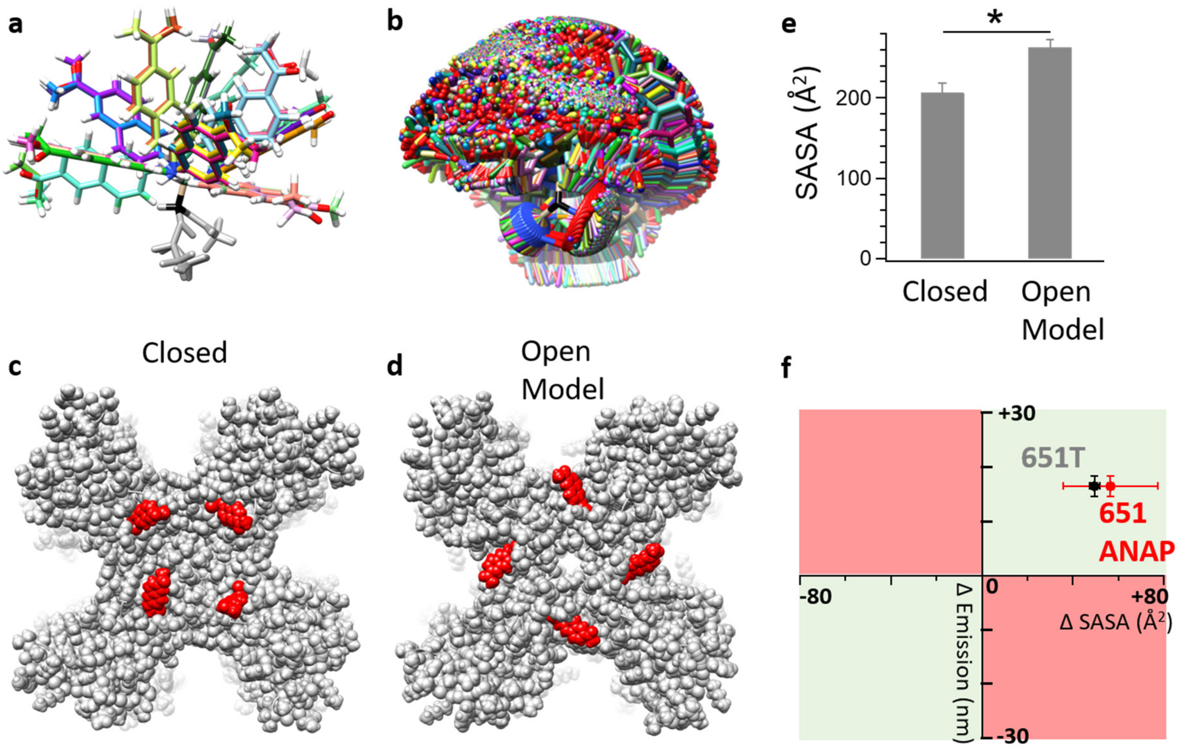

Supplementary Figure 4. Incorporation of ANAP into TRPV1 models. (**a**) A representative backbone dependent rotamer library of ANAP (phi and psi angle: -60° and -30°, respectively). Atoms in the backbone are colored in gray. (**b**) The full backbone dependent rotamer library. Phi and psi angle are binned from -180° to 180° with a 10° interval, respectively. (**c** and **d**) ANAP residue (red) was incorporated into TRPV1 models in the closed and open states, respectively. (**e**) SASA of ANAP at 651 site was significantly increased in the open state (*, $p < 0.05$; $n = 4$) (**f**) Correlation between shift in ANAP emission peak (y axis) and changes in SASA measured from cryo-EM structures (x axis) for the 651 site. When ANAP was incorporated at this site, it showed an increase in SASA similar to the increase in SASA measured for a Threonine residue in Fig. 3c. n = 5-to-7. All statistical data are given as mean ± s.e.m.

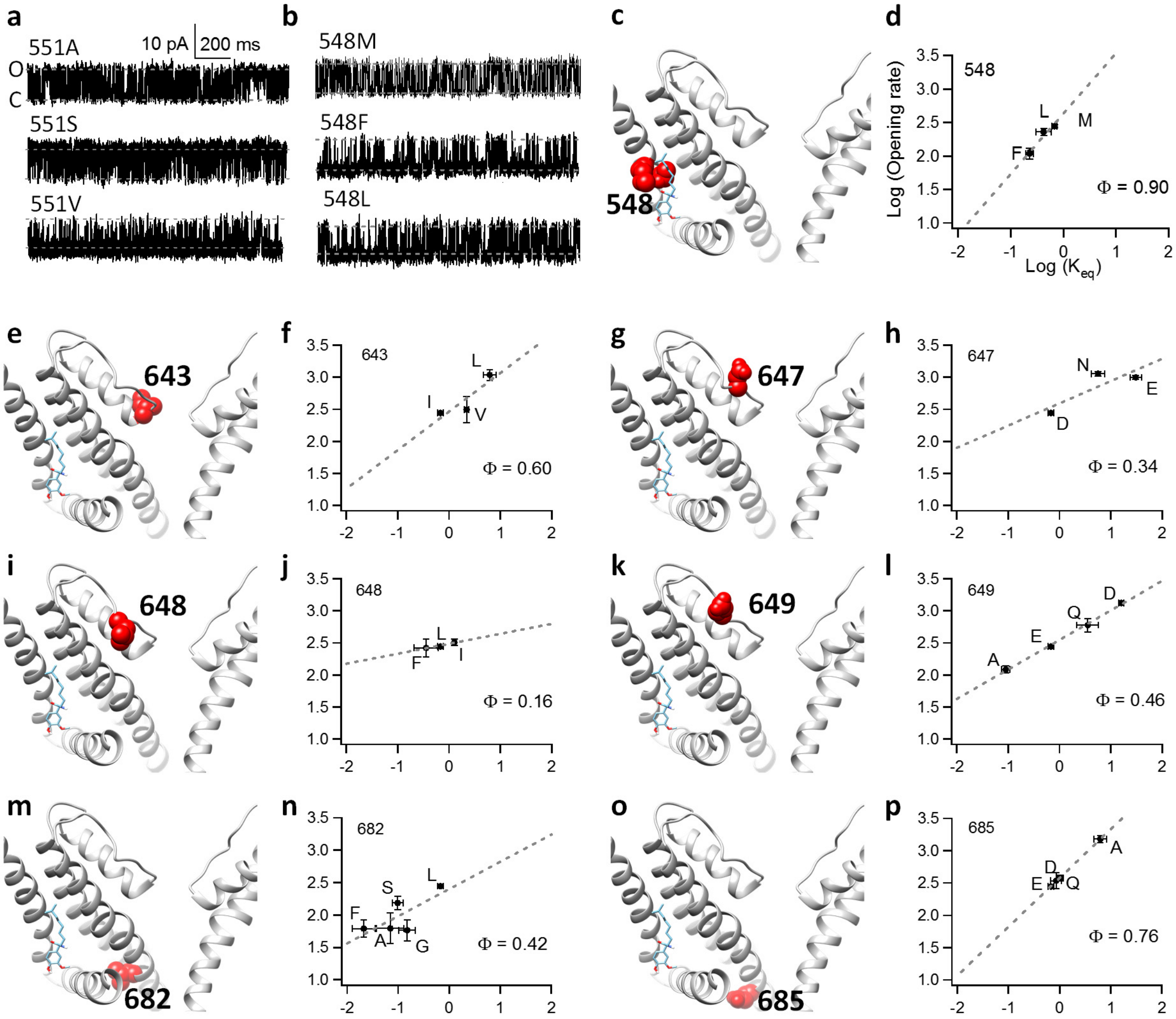

Supplementary Figure 5. Φ-analysis for additional sites on TRPV1. (**a** and **b**) Representative single-channel recordings of multiple mutations at the T551 and M548 sites, respectively. (**c**) M548 site (red) locates within the capsaicin binding pocket. (**d**) Brønsted plot to determine the Φ value for the 548 site. (**e** and **f**) Location and Brønsted plot to determine the Φ value for the 643 site. (**g** and **h**) Location and Brønsted plot to determine the Φ value for the 647 site. (**i** and **j**) Location and Brønsted plot to determine the Φ value for the 648 site. (**k** and **l**) Location and Brønsted plot to determine the Φ value for the 649 site. (**m** and **n**) Location and Brønsted plot to determine the Φ value for the 682 site. (**o** and **p**) Location and Brønsted plot to determine the Φ value for the 685 site.

Supplementary Table 1. Assessments of model quality.

	ERRAT	VERIFY_3D	WHAT_CHECK			PROVE
	Overall quality	Compatibility with sequence	Pass	Warning	Error	Outlier atoms
**TRPV1 (3J5P)**	86.213	67.47%	77	26	6	7.2%
**TRPV1 (5IRZ)**	63.375	35.79%	70	19	6	7.4%
**TRPV1 Open Model**	86.301	56.83%	74	26	5	6.0%

TRPV1 structures determined from cryo-EM, as well as open model of TRPV1 we computed with the Rosetta suite (shaded in green), were assessed by four independent protein structure analysis and verification methods (Green: higher in number is better; Orange and Red: lower in number is better).

Supplementary Table 2. **Primers used in this study to generate point mutations.**

Name	Primer-Forward	Primer-Reverse
D647A	GACACTGATCGAGTGTGGGAAGAATAACTC	GAGTTATTCTTCCCACACTCGATCAGTGTC
D647E	CACCATCGGCATGGGTGCCCTGGAGTTCAC	GTGAACTCCAGGGCACCCATGCCGATGGTG
D647Q	CACCATCGGCATGGGTGTCCTGGAGTTCAC	GTGAACTCCAGGACACCCATGCCGATGGTG
D647V	CTATCGGCCTGTGTGTGGCTTGCCCCCCTA	TAGGGGGGCAAGCCACACACAGGCCGATAG
E571A	TGCTGTCATGATTTGCAAGATGATCCTCAG	CTGAGGATCATCTTGCAAATCATGACAGCA
E571C	TGCTGTCATGATTGACAAGATGATCCTCAG	CTGAGGATCATCTTGTCAATCATGACAGCA
E571D	TGCTGTCATGATTCAGAAGATGATCCTCAG	CTGAGGATCATCTTCTGAATCATGACAGCA
E571Q	GTCATGATTAGGAAGATGATCCTCAGAGACC	GGTCTCTGAGGATCATCTTCCTAATCATGAC
E571R	GTGACACTGATCTGCGATGGGAAGAATAA	TTATTCTTCCCATCGCAGATCAGTGTCAC
E649A	TGGGTGACCTGGAGGCCACCGAGAACTATGA	TCATAGTTCTCGGTGGCCTCCAGGTCACCCA
E649D	TGGGTGACCTGGAGATCACCGAGAACTATGA	TCATAGTTCTCGGTGATCTCCAGGTCACCCA
E685A	TGGGTGACCTGGAGATGACCGAGAACTATGA	TCATAGTTCTCGGTCATCTCCAGGTCACCCA
E685D	TCGGCCTGTGGAATGTTTGCCCCCCTATA	TATAGGGGGGCAAACATTCCACAGGCCGA
E685Q	CACTGATCGAGGATTGCAAGAATAACTCAC	GTGAGTTATTCTTGCAATCCTCGATCAGTG
F650A	GCTGTCTTCATCGCCCTGTTACTGGCCTATG	CATAGGCCAGTAACAGGGCGATGAAGACAGC
F650A	TGCTCAACATGCTCGCTGCTCTCATGGG	CCCATGAGAGCAGCGAGCATGTTGAGCA
F650I	TGCTCAACATGCTCTTTGCTCTCATGGG	CCCATGAGAGCAAAGAGCATGTTGAGCA
F650I	TGCTCAACATGCTCGGTGCTCTCATGGG	CCCATGAGAGCACCGAGCATGTTGAGCA
F650L	TGCTCAACATGCTCCTTGCTCTCATGGG	CCCATGAGAGCAAGGAGCATGTTGAGCA
F650M	TGCTCAACATGCTCAGTGCTCTCATGGG	CCCATGAGAGCACTGAGCATGTTGAGCA
F650M	CTGATCGAGGATGGGTGCAATAACTCACTGC	GCAGTGAGTTATTGCACCCATCCTCGATCAG
F650Y	CTGGAGCTGTTCGAGTTCACCATCGGCATG	CATGCCGATGGTGAACTCGAACAGCTCCAG
G684A	GGACAGCTACAGTGAGATAGCTTTCTTTGTACAG	CTGTACAAAGAAAGCTATCTCACTGTAGCTGTCC
G684S	GCATGGGTGACGCGGAGTTCACCGAGAA	TTCTCGGTGAACTCCGCGTCACCCATGC
G684V	GGCATGGGTGACTTCGAGTTCACCGAGAA	TTCTCGGTGAACTCGAAGTCACCCATGCC
I574A	TGGCCTATGTGATTGCCACCTACATCCTC	GAGGATGTAGGTGGCAATCACATAGGCCA
I643A	CTACATCCTCCTGGCCAACATGCTCATTG	CAATGAGCATGTTGGCCAGGAGGATGTAG
I643L	CTACATCCTCCTGTGCAACATGCTCATTG	CAATGAGCATGTTGCACAGGAGGATGTAG
I643V	CTACATCCTCCTGTTCAACATGCTCATTG	CAATGAGCATGTTGAACAGGAGGATGTAG
I680A	CATGCTCATTGCTGCCATGGGCGAGACTG	CAGTCTCGCCCATGGCAGCAATGAGCATG
I680F	CATGCTCATTGCTTTCATGGGCGAGACTG	CAGTCTCGCCCATGAAAGCAATGAGCATG
I680G	CATGCTCATTGCTGGAATGGGCGAGACTG	CAGTCTCGCCCATTCCAGCAATGAGCATG
I680L	CATGCTCATTGCTAGCATGGGCGAGACTG	CAGTCTCGCCCATGCTAGCAATGAGCATG
I680S	TTCTCCCTGGCCTTCGGCTGGACCAACATG	CATGTTGGTCCAGCCGAAGGCCAGGGAGAA
L648A	GTTTGTGGACAGCTACACTGAGATACTTTTCTTTG	CAAAGAAAAGTATCTCAGTGTAGCTGTCCACAAAC

L648A	GTTTGTGGACAGCTACTATGAGATACTTTTC	GAAAAGTATCTCATAGTAGCTGTCCACAAAC
L648F	CAAGTGTCGGGGACCTGCCTGCAGGCCAG	CTGGCCTGCAGGCAGGTCCCCGACACTTG
L648F	GTAACTCTTACAACCCACTGTATTCCACATG	CATGTGGAATACAGTGGGTTGTAAGAGTTAC
L648G	GCCATGGGCTGGGCCAACATGCTCTACTAC	GTAGTAGAGCATGTTGGCCCAGCCCATGGC
L648G	GCCATGGGCTGGTCCAACATGCTCTACTAC	GTAGTAGAGCATGTTGGACCAGCCCATGGC
L648I	GCCATGGGCTGGGTCAACATGCTCTACTAC	GTAGTAGAGCATGTTGACCCAGCCCATGGC
L648V	CAGCCTGTATTCCCCATGTCTGGAGCTGTTC	GAACAGCTCCAGACATGGGGAATACAGGCTG
L676C	GTTTGTGGACAGCTGCAGTGAGATACTTTTC	GAAAAGTATCTCACTGCAGCTGTCCACAAAC
L676F	GTTTGTGGACAGCTTCAGTGAGATACTTTTC	GAAAAGTATCTCACTGAAGCTGTCCACAAAC
L676G	CATGGGTGACCTGGCGTTCACCGAGAACTA	TAGTTCTCGGTGAACGCCAGGTCACCCATG
L682A	CATGGGTGACCTGGACTTCACCGAGAACTA	TAGTTCTCGGTGAAGTCCAGGTCACCCATG
L682F	GTGACCTGGAGTTCTCCGAGAACTATGACTT<br>CA	TGAAGTCATAGTTCTCGGAGAACTCCAGGTC<br>AC
L682G	GTGACCTGGAGTTCCAGGAGAACTATGACTT<br>CA	TGAAGTCATAGTTCTCCTGGAACTCCAGGTC<br>AC
L682S	GTGACCTGGAGTTCTTGGAGAACTATGACTT<br>CA	TGAAGTCATAGTTCTCCAAGAACTCCAGGTC<br>AC
M548A	GCATGGGTGACGGGGAGTTCACCGAGAA	TTCTCGGTGAACTCCCCGTCACCCATGC
M548F	GGCATGGGTGACTTCGAGTTCACCGAGAA	TTCTCGGTGAACTCGAAGTCACCCATGCC
M548L	GGCATGGGTGACGTCGAGTTCACCGAGAA	TTCTCGGTGAACTCGACGTCACCCATGCC
M548Q	GGCATGGGTGACATCGAGTTCACCGAGAA	TTCTCGGTGAACTCGATGTCACCCATGCC
M645I	TGGGTGACCTGGAGGCCACCGAGAACTATGA	TCATAGTTCTCGGTGGCCTCCAGGTCACCCA
M645L	TGGGTGACCTGGAGATCACCGAGAACTATGA	TCATAGTTCTCGGTGATCTCCAGGTCACCCA
M645V	TGGGTGACCTGGAGATGACCGAGAACTATGA	TCATAGTTCTCGGTCATCTCCAGGTCACCCA
M683F	TGGGTGACCTGGAGTACACCGAGAACTATGA	TCATAGTTCTCGGTGTACTCCAGGTCACCCA
M683L	TGGGTGACCTGGAGCTGACCGAGAACTATGA	TCATAGTTCTCGGTCAGCTCCAGGTCACCCA
M683T	CATCGGCATGGGTGAGCTGGAGTTCACCGA	TCGGTGAACTCCAGCTCACCCATGCCGATG
T551A	GTTCACCATCGGCATCGGTGACCTGGAGTTC<br>A	TGAACTCCAGGTCACCGATGCCGATGGTGAA<br>C
T551S	CTCATTGCTCTCTTCGGCGAGACTGTCAA	TTGACAGTCTCGCCGAAGAGAGCAATGAG
T551V	CTCATTGCTCTCCTGGGCGAGACTGTCAA	TTGACAGTCTCGCCCAGGAGAGCAATGAG
T642A	CATTGCTCTCATGGCCGAGACTGTCAACAA	TTGTTGACAGTCTCGGCCATGAGAGCAATG
T642G	CATTGCTCTCATGGTCGAGACTGTCAACAA	TTGTTGACAGTCTCGACCATGAGAGCAATG
T642L	CATTGCTCTCATGAGCGAGACTGTCAACAA	TTGTTGACAGTCTCGCTCATGAGAGCAATG
T642M	TTGCTCTCATGGGCGACACTGTCAACAAGA	TCTTGTTGACAGTGTCGCCCATGAGAGCAA
T642N	TTGCTCTCATGGGCCAGACTGTCAACAAGA	TCTTGTTGACAGTCTGGCCCATGAGAGCAA
T642S	TTGCTCTCATGGGCGCGACTGTCAACAAGA	TCTTGTTGACAGTCGCGCCCATGAGAGCAA
T651L	CTCTCATGGGCGAGTCTGTCAACAAGATTG	CAATCTTGTTGACAGACTCGCCCATGAGAG
T651Q	CTCTCATGGGCGAGCTTGTCAACAAGATTG	CAATCTTGTTGACAAGCTCGCCCATGAGAG
T651S	CTCTCATGGGCGAGAATGTCAACAAGATTG	CAATCTTGTTGACATTCTCGCCCATGAGAG

T686L	CATGCTCTACTACACCGAAGGATTCCAGCAGA	TCTGCTGGAATCCTTCGGTGTAGTAGAGCATG
T686N	CATGCTCTACTACACCGACGGATTCCAGCAGA	TCTGCTGGAATCCGTCGGTGTAGTAGAGCATG
T686S	CATGCTCTACTACACCAACGGATTCCAGCAGA	TCTGCTGGAATCCGTTGGTGTAGTAGAGCATG
C579_TAG	AGACGAACATAAACCGCTACAGGTCTCTGAGGATCATCTTC	GAAGATGATCCTCAGAGACCTGTAGCGGTTTATGTTCGTCT
T651_TAG	GCCTTGAAGTCATAGTTCTCCTAGAACTCCAGGTCACCCATGC	GCATGGGTGACCTGGAGTTCTAGGAGAACTATGACTTCAAGGC
Y654_TAG	GACAGCCTTGAAGTCCTAGTTCTCGGTGAACTCC	GGAGTTCACCGAGAACTAGGACTTCAAGGCTGTC

Supplementary Methods

Commands in Rosetta to perform loop modeling:

```
/home/fanyang/rosetta/main/source/bin/loopmodel.linuxgccrelease \
-in:path:database /home/fanyang/rosetta2015.25/main/database \
-score:weights membrane_highres_Menv_smooth.wts \
-in:file:fullatom \
-membrane:normal_cycles 100 \
-membrane:normal_mag 15 \
-membrane:center_search \
-ignore_unrecognized_res \
-symmetry:symmetry_definition
/home/fanyang/projects/input_files/3J5R_2017/4D_ABCD_r_4D_after_ccd_relaxed_r.symm \
-symmetry:initialize_rigid_body_dofs \
-in:file:spanfile /home/fanyang/projects/input_files/3J5R_2017/3J5R_FL.span \
-in:file:s /share/work/fanyang/work/3J5R_FL_KIC5-3J5R_2017-
_/top20_score_filtered/_1D/${SLURM_ARRAY_TASK_ID}.pdb \
-loops:loop_file /home/fanyang/projects/input_files/3J5R_2017/3J5R_FL_SF.loop \
-loops:remodel perturb_kic \
-loops:refine refine_kic \
-loops:relax no \
-loops:strict_loops \
-loops:build_attempts 20 \
-relax:bb_move false \
-max_inner_cycles 30 \
-nstruct 51 \
-out:prefix msymm-loop-kic- \
```

```
-out:file:silent /share/work/fanyang/work/3J5R_FL_KIC6-3J5R_2017-
_/${SLURM_ARRAY_TASK_ID}/msymm-relax-_3J5R_2017_${SLURM_ARRAY_TASK_ID}.silent \
-out:file:silent_struct_type binary \
-mute all
```

Commands in Rosetta to perform SASA calculation and filtering:

```
/home/fanyang/rosetta/main/source/bin/rosetta_scripts.linuxgccrelease \
-database /home/fan/rosetta_2016.20/main/database
-membrane:normal_cycles 100
-membrane:normal_mag 15
-membrane:center_search
-in:file:spanfile
/media/fan/Data/Rosetta/Project_3J5R_loopmodeling2017/inputs_fullLength/3J5R_FL.span
-score:weights membrane_highres_Menv_smooth.wts
-parser:protocol
/media/fan/Data/OneDrive/Academic/Academic_Computation/Rosetta_script/xml_scripts_Fan
/filter_sasa_FY_T651.xml
-symmetry:symmetry_definition
/media/fan/Data/Rosetta/Project_3J5R_loopmodeling2017/inputs_fullLength/4D_ABCD_r_4D_
3J5R_FL_r.symm
-symmetry:initialize_rigid_body_dofs
-ignore_unrecognized_res
-in:file:silent_struct_type binary
-in:file:silent
/media/fan/Data/Rosetta/Project_3J5R_loopmodeling2017/FL_after_KIC4/3J5R_FL_KIC3-
3J5R_2017-_SILENT.out
-nstruct 1
-out:file:silent
/media/fan/Data/Rosetta/Project_3J5R_loopmodeling2017/FL_after_KIC4/Filtered_T651_aft
erKIC4.silent
-out:file:silent_struct_type binary
-overwrite
```

Rosetta scripts to perform SASA calculation and filtering:

```
<ROSETTASCRIPTS>
    <RESIDUE_SELECTORS>
      <Not name="T651_unselected">
            <Index resnums=293/>
      </Not>
    </RESIDUE_SELECTORS>
    <TASKOPERATIONS>
      <OperateOnResidueSubset name="T651_only" selector="T651_unselected" >
            <PreventRepackingRLT/>
      </OperateOnResidueSubset>
    </TASKOPERATIONS>
    <SCOREFXNS>
    </SCOREFXNS>
 <FILTERS>
      <TotalSasa name=T651_sasa threshold=10 task_operations=T651_only
report_per_residue_sasa=1/>
 </FILTERS>
    <MOVERS>
    </MOVERS>
    <PROTOCOLS>
      <Add filter=T651_sasa/>
    </PROTOCOLS>

</ROSETTASCRIPTS>
```

Commands in Rosetta to perform global refinement and FSC calculation:

```
-database /home/fan/rosetta/main/database

-in::file::s /media/fan/Data1/Rosetta/Project_3J5R_loopmodeling2017/relax_cryoEM/3J5R.pdb

-parser::protocol /media/fan/Data1/Rosetta/Project_3J5R_loopmodeling2017/relax_cryoEM/B_relax_density.xml

-parser::script_vars reso=4.2 map=/media/fan/Data1/Rosetta/Project_3J5R_loopmodeling2017/relax_cryoEM/EMD-5777_CAP_3j5r.map testmap=/media/fan/Data1/Rosetta/Project_3J5R_loopmodeling2017/relax_cryoEM/EMD-5777_CAP_3j5r.map

-ignore_unrecognized_res

-default_max_cycles 200

-edensity::cryoem_scatterers

-out::suffix cryoEM_Relax_

-out:file:silent /media/fan/Data1/Rosetta/Project_3J5R_loopmodeling2017/relax_cryoEM/CAP_OpenModel_cryoEM_Relaxed.silent

-out:file:silent_struct_type binary

-nstruct 1

-overwrite
```

Rosetta scripts to perform global refinement and FSC calculation:

```
<ROSETTASCRIPTS>
   <SCOREFXNS>
      <dens weights=talaris2013_cart>
         <Reweight scoretype=elec_dens_fast weight=20.0/>
      </dens>
   </SCOREFXNS>

   <MOVERS>
       <SetupForDensityScoring name=setupdens/>
       <LoadDensityMap name=loaddens mapfile="%%map%%"/>
       <ReportFSC name=report testmap="%%testmap%%" res_low=10.0 res_high=%%reso%%/>
       <FastRelax name=relaxcart scorefxn=dens repeats=1 cartesian=1/>
   </MOVERS>

   <PROTOCOLS>
      <Add mover=setupdens/>
      <Add mover=loaddens/>
      <Add mover=relaxcart/>
      <Add mover=report/>
   </PROTOCOLS>
   <OUTPUT scorefxn=dens/>
</ROSETTASCRIPTS>
```

Commands in Rosetta to incorporate ANAP into TRPV1 models

```
-database /home/fan/rosetta/main/database
-in:file:fullatom
-ignore_unrecognized_res
-s /home/fan/Rosetta/Project_ANAP_Parameterization/3J5P.pdb
-backrub:ntrials 10
-nstruct 1
-mc_kt 0.6
-resfile /home/fan/Rosetta/Project_ANAP_Parameterization/T651ANP_3J5P.resfile
-overwrite
```

T651ANP_3J5P.resfile:

```
NATRO
start
651 A EMPTY NC ANP
651 B EMPTY NC ANP
651 C EMPTY NC ANP
651 D EMPTY NC ANP
```
