# Supplementary material for: The conformational wave in capsaicin activation of transient receptor potential vanilloid 1 ion channel
Source: Nat Commun. 2018 Jul 23;9:2879. doi: 10.1038/s41467-018-05339-6 (PMC6056546; doi:10.1038/s41467-018-05339-6)
Supplement: Supplementary file 3 — Description of Additional Supplementary Files [file 41467_2018_5339_MOESM3_ESM.docx]

**Description of Additional Supplementary Files**

File Name: Supplementary Data 1

Description:

Three clusters of TRPV1 capsaicin-bound open state models with top 10 lowest-energy values. This .py file is opened by the software UCSF Chimera. Cluster 1, 2 and 3 are colored in red, orange and purple, respectively.

File Name: Supplementary Data 2

Description:

The representative TRPV1 capsaicin-bound open state model. This is the final open state model shown in Figure 3b and 3d (colored in red).

File Name: Supplementary Data 3

Description:

The rotamer library of ANAP. This rotamer library is used in Rosetta to determine the sidechain conformation of ANAP when incorporated at the 651 site of TRPV1 (Supplementary Figure 4).

File Name: Supplementary Data 4

Description:

The TRPV1 capsaicin-bound open state model with ANAP incorporated at the 651 site.

File Name: Supplementary Data 5

Description:

The apo state TRPV1 model with ANAP incorporated at the 651 site.
